# Effect of Water Regeneration and Integration on Technical Indicators of PVC Manufacturing Using Process System Engineering

**DOI:** 10.3390/polym17172418

**Published:** 2025-09-06

**Authors:** Eduardo Andrés Aguilar-Vásquez, Segundo Rojas-Flores, Ángel Darío González-Delgado

**Affiliations:** 1 Nanomaterials and Computer Aided Process Engineering Research Group (NIPAC), Universidad de Cartagena, Chemical Engineering Department, Cartagena de Indias 130015, Colombia; eaguilarv@unicartagena.edu.co; 2 Institutos y Centros de Investigación, Universidad Cesar Vallejo, Trujillo 13001, Peru; srojasf@ucv.edu.pe

**Keywords:** mass integration, regeneration, WEP analysis, sustainability, PVC, process system engineering

## Abstract

The suspension polymerization process of polyvinyl chloride (PVC) production involves significant freshwater consumption alongside substantial wastewater emissions. Mass integration strategies have been used to address this problem, but only through direct recycling approaches. Therefore, in this study, a regeneration approach was applied to integrate a PVC suspension process to improve water management. The reuse network was evaluated through a water–energy–product (WEP) technical analysis after being simulated in AspenPlus software v.14. The mass integration allowed for a 61% reduction in freshwater consumption and an 83% reduction in wastewater. However, 258.6 t/day of residual wastewater still remained after regeneration. The WEP analysis found that the process was efficient in handling raw materials and process products due to the high yield and recovery of unreacted materials. Similarly, the integration significantly benefitted the process performance as water usage indicators improved substantially, with freshwater consumption of 83%, a wastewater production rate of 63%, and freshwater water costs of $267,322 per year (from $694,080 before integration). In terms of energy performance, the results were regular. The processes showed high energy consumption (below 50%), with indicators related to the use of natural gas, electricity, and energy costs being affected by the regeneration. However, the limited heat integration provided minor energy savings (11 MJ/h). Finally, this work gives an interesting insight into water conservation and the circular economy, since the study used the latest systems in regeneration of effluents for plastic plants (emerging technologies), showcasing important benefits and trade-offs of these strategies.

## 1. Introduction

The UN projects that the global water demand will increase from 3500 km^3^ per year in 2000 to around 5500 km^3^ per year by 2050. Additionally, several studies estimate that the share of the industrial and energy sectors in global water demand will grow to 24% by 2050 [1]. Since water is a key element for the normal functioning of the manufacturing industry [2], being widely used in chemical, petrochemical, food and beverage, pulp and paper, and textile industries, among many others. However, the improper use of water is a critical issue in all regions of the planet. The imbalance between demand and supply has caused continuous scarcity, which not only threatens processing facilities but also impacts economic, social, and ecological aspects. As a result, the United Nations’ Sustainable Development Goals (SDGs) call for a change from industries, governments, society, and individuals to reach adequate levels of awareness and action in response to sustainability challenges [3]. Stricter environmental regulations and the scarcity of quality industrial water have pushed the chemical industry to reduce water usage by adopting conservation strategies such as water reuse, recycling, and regeneration [4], promoting sustainable, socially responsible water consumption (in compliance with regulations) and environmentally respectful practices.

Process integration provides a useful framework for developing sustainable processes based on resource conservation principles [5]. Within this concept, process mass integration is a powerful tool that allows for the conservation of mass resources, maximizing economic performance and minimizing environmental impacts [6]. This techniques helps to fundamentally understand the global mass flow within processes, identifying objectives that enable the minimization of resource consumption and waste emissions [7]. For the implementation of this systematic methodology, numerous methods exist, including graphical and mathematical approaches, such as algebraic and mathematical programming [8]. Additionally, minimization can be achieved through various strategies such as direct recycling or reuse after a regeneration stage, involving operations such as stream mixing, splitting, recycling, the use of separation devices, changes in operational parameters, and the design of separation units, among others [9].

The use of indicators has become an essential tool for achieving sustainable production, due to the impacts of mass and energy consumption that chemical processes have on the economy, society, and the environment [10]. These indicators facilitate comparative analyses and the tracking of performance objective fulfillment [11]. Nonetheless, the effectiveness of the indicators is conditioned by the availability of information about the analyzed process. The application of process systems engineering (PSE) allows for understanding the operation of process systems at multiple scales [12]. Tools such as those related to modeling and simulation enable the analysis and prediction of process behavior before industrial implementation, offering reliable results to support decision-making [13].

Polyvinyl chloride (PVC) is part of the group of commodity plastics, being the third most produced plastic [14]. This polymer is characterized by its high versatility and durability [15]. PVC production has recently plateaued or shown slight declines in mature markets but remains stable overall. Suspension polymerization still dominates ~80% of the global output, while non-suspension processes now contribute ~10–12% (emulsion and bulk methods). Although suspension PVC is cheaper and more efficient per ton, emulsion and micro-suspension technologies are growing in importance, especially for specialty, flexible, or “eco-improved” applications. These methods can reduce energy and water use and allow a finer resin morphology, making them appealing in regions with tightening environmental regulations. Suspension polymerization presents an energy consumption range of 0.7–1.1 GJ/t and VCM emissions of 80–100 g/t; on the other hand, emulsion polymerization presents an energy consumption range of 1.4–2.2 GJ/t and VCM emissions of 500–900 g/t. As sustainable manufacturing gains momentum, non-suspension methods—especially improved emulsion and micro-suspension processes—are expected to capture a larger fraction of small but high-value markets over the next decade [16]; in suspension polymerization, the vinyl chloride monomer (VCM) polymerizes within a water suspension with additives (initiator, suspension agent, and others) [17]. This production method is known for its high productivity and flexibility in terms of the polymer characteristics [18]. However, the process presents significant sustainability challenges, such as intensive resource consumption (water and energy), the emission of toxic substances, health risks, greenhouse gas emissions [16], and low reuse, among others [19]. The water management of this process is a major challenge; reducing water intake across various stages is a key performance target, with the reaction stage being a priority. The formation of the suspension required for polymerization involves significant volumes of demineralized water. Additionally, the use of additives necessary to ensure product quality contributes to the formation of large volumes of wastewater, which are expelled during the drying stage, in the form of centrifuge water. This flow represents the main source of effluents in the process. Therefore, the implementation of process integration techniques is a promising option to minimize water consumption in the process.

Due to the significant water footprint of the suspension process for PVC production, multiple studies have been conducted to explore potential savings in the process through mass integration of water resources, as shown in Table 1. These studies present a variety of considerations, including process boundaries such as sources (wastewater-producing units) and sinks (water-using units), identified critical pollutants (e.g., from process and utilities equipment or other product’s processes), the methodology employed, and the main objective or target, among others.

In Table 1, the main similarity of the previous studies is the use of direct recycling or reuse approaches, as Chan et al. applied integration techniques for a PVC process through direct recycling (without regeneration). They also considered the batch operation of the processes when aiming to minimize consumption, such as the reactor or the dewatering units; they included units from the utilities systems (boiler and cooling tower makeup water), along with water for multiple services as sinks. The techniques were effective in reducing wastewater discharge (90.1%) and achieving acceptable minimization of freshwater (31.7%). It was found that the operation time and the number of reactor cycles influenced the water sink flows and the flows returned to the system. Additionally, storage allows for maximizing water recycling [20]. Similarly, Zheng et al. integrated a plant producing sodium hydroxide and PVC. For this, several factors were considered regarding the permissible limit of contaminants for the studied units (equipment). At least 11 sinks were considered, including operational and utilities units (including water for multiple services). The results showed that the composition is a limiting factor for designing the effluent distribution, whereby 26% savings and a 48% reduction in wastewater were achieved. Additionally, it was found that the wastewater from the PVC process is complex to recirculate due to its composition (organic compounds), with decentralized regeneration being an alternative that can increase wastewater reuse [21]. On the other hand, Lee et al. combined production scheduling and mass integration through linear programming using GAMS software to minimize water consumption (flow and cost). The reactor and cleaning water were considered as sinks. Suspended solids were considered the main contaminant. A reduction of 32,720 tons per year of freshwater was achieved, with a minimum cost of 65,267 per year. Additionally, a storage tank of 52,667 tons is needed to store wastewater with 50 ppm [22].

The regeneration approach in water minimization entails the partial or total treatment of wastewater for further use (increase in quality) to minimize water consumption. Skouteris et al. compared both strategies in a brick manufacturing plant, finding that direct recycling and reuse strategies shown low savings of 15.6%, while the regeneration approach achieve 80.4% savings when systematically implemented (optimal) [23]. Something similar was found by Espindola et al., when analyzing strategies to reduce the freshwater intake of a dairy plant, as having several robust regeneration systems available can offer a higher number of potential alternatives networks that can increase water savings, as more contaminants are treated efficiently; a combined membrane bioreactor (MBR) and nanofiltration achieved 69.5% savings compared to 46% of the MBR alone with direct recycling and reuse [24]. The use of regeneration systems also allows the exploration of centralized and decentralized systems, as multiple contaminants are commonly found in industrial wastewater with different effects on the sinks and the regeneration technology, as Esmaeeli et al. found when implementing a water closed-loop system (WCLS) in a pulp and paper plant [25]. Nonetheless, higher savings is not a synonym for viability, as economic and technical factors can limit the application of these strategies. Authors such as Wan Alwi et al. state that regeneration should be a final approach to minimize the water footprint, as it is a high-impact, high-cost approach [26].

The analyzed studies show limitations of the direct reuse and recycling approach when integrating the PVC suspension process. The composition of sources and the restrictive quality requirements of the sinks are the main limiting factors that hinder wastewater utilization. As regeneration offers higher savings due to effluent quality improvements, an important knowledge gap exists, as there is a scarcity of information related to the implementation of integration processes using regeneration for PVC production via suspension, opening an opportunity to explore the gains and drawbacks of this approach. Therefore, this work presents a mass integration method with regeneration aiming to minimize water consumption in an industrial-scale PVC suspension production process (thermally integrated). Additionally, this work offers a methodology that combines process systems engineering tools with a technical evaluation to provide a broader assessment beyond an economic perspective to assess the effect of the integration on the resource efficiency of the process. Furthermore, the technical evaluation identifies bottlenecks, potential savings, trade-offs, and opportunities for improvement that ultimately enhance the sustainability of PVC production.

## 2. Materials and Methods

**Figure 1:**
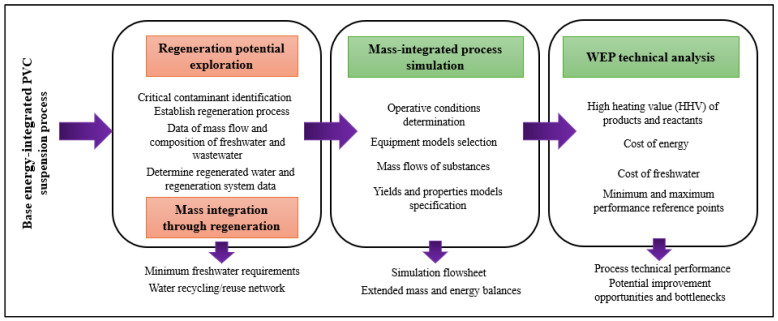
Methodology framework for performing this research.

Figure 1 details the scheme of the methodology used for integrating the PVC suspension process. A methodology similar to that described by Moreno-Sader et al. is employed, combining mass integration with a process analysis method using simulation software [27]. A base industrial-scale PVC suspension production process is studied, and the graphical method of the sink–source map is applied with the goal of minimizing the freshwater requirements and residual water effluents of the process. Based on the results obtained from the integration, a process simulation is constructed using specialized software (AspenPlus V14). Considering preliminary flows, thermodynamic information, units or equipment, etc., the extended mass and energy balances generated by the simulator are used to collect the operational process information needed to calculate the technical indicators. These indicators allow the diagnose of the process using the water–energy–product (WEP) technical analysis methodology [28], which provides feedback on performance in terms of efficient resource use and identifies opportunities for optimization.

### 2.1. Description of the Energy-Integrated Suspension PVC Production Process

**Figure 2:**
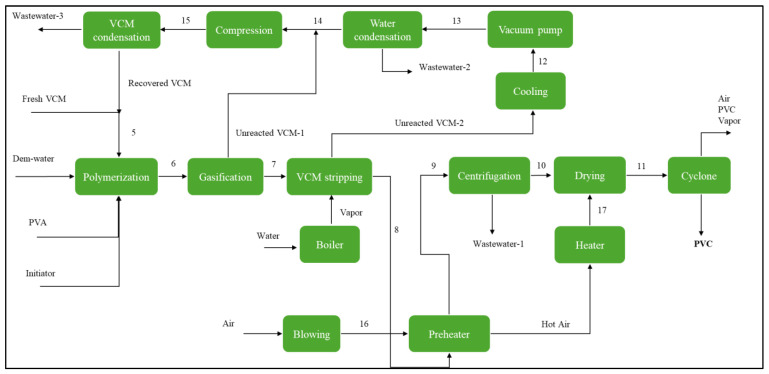
Flow diagram of the energy-integrated PVC suspension process.

In Figure 2, the flow diagram of the energy-integrated industrial-scale suspension PVC production process is shown. This process is based on studies performed previously by the authors of [29] and begins with the polymerization reactor system, where liquid VCM (fresh and recirculated) is converted into PVC in a water (demineralized) suspension with the presence of a polymer initiator (3-hydroxy-1,1-dimethylbutane-2-ethyl-2-methylheptane peroxide) and a suspension stabilizer (PVA). The reaction takes place at a temperature of 70 °C and a pressure of 10 kg-f × cm^−2^, with a conversion rate of around 85%. At the end of the reaction, a heterogeneous mixture remains, containing the suspended polymer, unreacted monomer, water, initiator, and stabilizer. This mixture exists at a pressure level of 3.5 kg-f × cm^−2^ and a temperature of around 70 °C.

Due to (highly toxic) emissions regulations, the unreacted VCM needs to be removed from the slurry after the reaction (less than 1 ppm). For the VCM purge, a flash tank and an absorption tower are used. The gasification stage removes 95% of the monomer by reducing the pressure to 1.8 kg-f × cm^−2^ (unreacted VCM-1). The volatility of the unreacted monomer is used to separate it from of the suspension. The remaining monomer (5%) is removed through a stripper column (unreacted VCM-2). This consists of a tray tower in which a steam flow at high pressure (14 kg-f × cm^−2^) and temperature (225 °C) extracts the VCM out of the slurry in a countercurrent. From the column, two streams are produced, one “rich” stream of monomer at the top and the purged slurry at the bottom with less than 1 ppm of VCM. The rich stream from the tower and the gas stream from the gasification stage enter the residual monomer recovery system, which comprises several heat exchangers and compressors that render the residual VCM suitable for its recirculation. The final stream has a pressure of 3.5 kg-f × cm^−2^, is in liquid state (8 °C), and is free of residual water.

The purged slurry from the stripper includes a large volume of water (approximately 70%) that must be removed. Before entering the drying stage, the potential damage to the PVC resin by the heating is reduced by energetically integrating it with the air stream to lower its temperature (below 70 °C). This stream then enters a centrifuge that spins at 1800 rpm, where around 75% of the suspension water is separated.

The wastewater from the centrifugation contains fractions of the polymer, almost all of the PVA, and the initiator. After dewatering, a wet paste consisting mainly of PVC is formed, which requires further drying using a rotary dryer. In this process, an air stream is heated in two stages via a heat exchanger (integration) and a burner, raising the temperature up to 250 °C. The dryer operates at a temperature of 70 °C. The final moisture content of the polymer is 0.01% by weight upon exiting the dryer [30]. Immediately, the dry PVC resin enters a cyclone to be separated from the gas mixture. It operates under atmospheric pressure conditions (1.03 kg-f × cm^−2^). The top stream still contains polymer particles (0.2% of the total produced) alongside air and water vapor, while the bottom stream is the dried polymer.

### 2.2. Mass Integration of Processes and Water Regeneration

For this work, the graphical method of source–sink mapping developed by El-Halwagi was used [31], as it allows for better visualization of the system and helps to pinpoint recycling opportunities. The main objective is to minimize the water consumption of the process, by using a mixed approach of reuse–recycling focused on water regeneration [32].

First, in the base case, potential sources and sinks of the process are identified, with the latter corresponding to process units that consume freshwater and the former being flows of water categorized as waste. Next, data such as those related to flow and mass composition are gathered for both the wastewater streams and for the sinks (water feed). Simultaneously, the critical contaminant is determined, which is the substance that exerts the greatest influence on the normal functioning of the sinks within the process.

For each wastewater stream and potential sink, a name is assigned ($s_{r}$ for sources and $s_{k}$ for sinks), and both are plotted considering the amount of limiting contaminant and the mass flow. More specifically, for the sinks, they are plotted according to the range of restrictions for the contaminant (maximum and minimum). The diagram is built by plotting the mass flow versus contaminant composition.

Using the “arm–lever” rule, mixed streams (with fresh source) are created to reuse or recycle wastewater streams according to the limitations of each sink. For each sink, the highest allowable range for the contaminant is used, and the smallest amount of flow necessary is considered (fresh source). The order of minimization of the sources follows the prioritization rules developed by the arm–lever rule, where it is stated that the order is ascending, starting with the source with the smallest fresh arm until it is fully recycled or no longer possible according to the sink’s limitations. If the source is depleted before the sink, the next nearest source is linked until the source is satisfied. This process is repeated for all sources until they are minimized.

Equation (1) is used to calculate the fresh requirement of a specific sink i:

(1) $$\dot{M_{fresh}}=\dot{M_{z}}\times \frac{y_{i}-z_{max}}{y_{i}-z_{min}}$$

where $y_{i}$ is the composition of the key contaminant of the source; $z_{max}$ and $z_{min}$ are the maximum and minimum allowable concentrations of the sink, respectively; $\dot{M_{z}}$ is the maximum flow allowed by the sink. The amount of reused material, $\dot{M_{r}}$, is calculated by subtracting the fresh resource flow obtained from the maximum flow of the sink, as Equation (2) shows.

(2) $$\dot{M_{r}}=\dot{M_{z}}-\dot{M_{fresh}}$$

A reduction in freshwater streams is the equivalent of a reduction in the overall wastewater streams. The total wastewater of the process is the residual flow left, $\dot{G_{w}}$, after linking, which is calculated by subtracting the reused or recycled flow, $\dot{M_{r}}$, from the total wastewater available to utilize, $\dot{G_{i}}$.

(3) $$\dot{G_{w}}=\dot{G_{i}}-\dot{M_{r}}$$

For this, both the maximum composition and the maximum mass flow of each source and sink are plotted. The composition is plotted on the *x*-axis, and the flow is plotted on the *y*-axis.

#### 2.2.1. Mass Integration Using Regeneration (Interceptor)

As stated early in this section, a regeneration approach was selected to maximize water use. For this, a regeneration or treatment (interceptors) system is chosen to enhance the streams’ quality to further increase reuse and recycling. These units have significant effects on processes, as while they maximize the utilization of residual streams, they may also incur additional costs [33].

To carry out the integration, several considerations must be made. Firstly, only the process streams involved in the production of the resin are considered, meaning no streams from the utilities systems are integrated. Second, the integration is carried out using a single contaminant.

The regeneration of effluents is performed prior to the reuse and recycling, so the integration of all streams is performed after the rectification of the selected stream(s).

Moreover, only one regeneration system is employed, which is selected by meeting two conditions:

(1) The output concentration of the treated streams should be significantly lower than the original concentration of the selected contaminant, $c_{out}\ll c_{in}$;
(2) An approximate fixed removal ratio or efficiency, $R=\frac{f_{in}*c_{in}-f_{out}*c_{out}}{f_{in}*c_{in}}$, or a percentage yield (%) is required.

All the water is treated before integration; no partial regeneration approach is considered. Both the output concentration and mass flow of the regenerated water are fixed.

#### 2.2.2. Simulation of the Water Energy and Water Integrated Process

The proposed integrated process is simulated through the AspenPlus v.14 software. This diagram was constructed based on previous studies by the authors of [28] and information from the literature (for the regenerating system). For the reuse of streams, the Broyden method was used to achieve the convergence of the simulation units. A 100% recycling rate was implemented for each stream. For the selected interceptor system, ideal models were used to reproduce the operations and reactions (e.g., reactors such as black boxes), according to the literature.

#### 2.2.3. Technical Analysis of the Energy- and Water-Integrated Process

The process analysis is carried out using the WEP methodology previously developed by the authors of this work [28]. This involves the quantification of indicators based on the extended mass and energy balances generated from the simulator, as seen in Table 2. The quantification of these indices provides a diagnosis of the performance of the efficiency of the material and energy use, as well as the costs. The performance is quantified using a benchmarking approach using performance reference points (best and worst values) and the normalization of these values. For this work, the same indicators and benchmark points from the aforementioned study were used, with the inclusion of performance reference points for the NER, with 1 and 0.5 as the best and worst cases, respectively.

## 3. Results and Discussion

### 3.1. Results of Mass Integration with Water Regeneration for PVC Production

Table 3 shows the consolidated sources identified in the PVC suspension process. Due to the small number of wastewater sources, the current streams from the condensers in the VCM recovery area are selected alongside the centrifuge wastewater. The identified sources show a system where water is mainly used as a transfer agent with fixed characteristics, i.e., both the load and flow [34]. The vapor stream discharge from the cyclone was not considered because it is in the gaseous phase, and additional energy exchange equipment is not desired in the process (unless strictly necessary). Likewise, the water contained in the product stream was not recoverable, as was required for the resin specifications (quality).

Polyvinyl alcohol (PVA) was identified as the key contaminant in the process; this compound represents the main obstacle for the reuse of process effluents due to its capacity to interfere in the suspension formation when accumulated; this effect is enhanced by its properties, which make it have a strong affinity with water, tending to be in the liquid phase rather than the solid phase [35].

The main challenge of this system is to adequately identify the sinks and restrictions. Table 4 presents the main sinks from the energy-integrated process, where the reactor is a major sink that represents 75% of the water consumption in the process. Additionally, the boiler for steam generation for the stripping column is another important sink. Furthermore, it is possible to replace the water used to form the PVA and fresh initiator solutions in the process, but the composition of both solutions represents a determining parameter for the quality of the final product, so they are not considered. The restrictions based on composition were assumed based on the information from Blanco et al. [36].

Moreover, several additional sinks exist, as boundaries of the integration process can be expanded to include water sinks in utilities systems, such as water for multiple purposes (e.g., wash or for fires, etc.) or makeup water for cooling towers. Both are considered as potential final sinks but were not included because they do not have limitations on contaminant composition (the input can be of lesser quality than the source used in the reactor) and are outside the internal process battery [37]. In the case of makeup water, it has a flow of 816 m^3^/day, and the water with multiple purposes accounts for 5% of the total water used in the plant; however, neither of these are considered in the reuse system.

Equations (1)–(3) were used, considering the effect of the interception unit on source 3, as it is the only stream with a concentration critically higher than the maximum allowable for each sink. The stream is regenerated by adjusting its initial composition to a more suitable one to maximize its reuse. PVA is a difficult material to remove using conventional methods, due to its refractory organic nature and its ability to interfere with membranes, in addition to its high foam formation [38]. Therefore, for the regeneration unit, a system composed of membrane bioreactors and reverse osmosis separators was selected, as found in the studies by Blanco et al., Prieto et al., and Kang [39,40]. Biological methods are very efficient, with yields ranging from 83% to 98%, depending on the microbial species [41]. Additionally, these methods can treat large volumes of wastewater with high concentrations of PVA [42].

**Figure 3:**
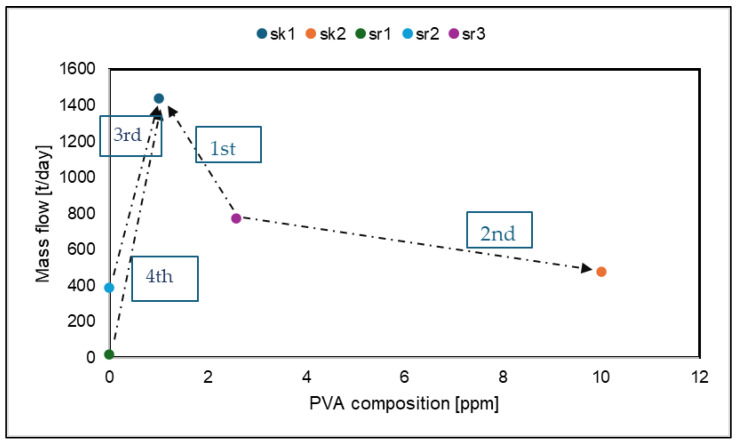
Sink–source map for the PVC suspension process.

Figure 3 shows the source–sink map for the PVC production process integrated via regeneration. The minimization order is shown for the considered sources. First, the source sr3 is placed, considering its characteristics after regeneration. The stream reaches a flow rate of 771 t/day, with a composition of 2.53 mgL^−1^, leaving a rejected concentrated wastewater stream of 258.6 t/day (sent for treatment as effluent to be discharged). The other sources and sinks are then positioned. In this figure, sr3 has a shorter arm to sk1 compared to sr1 and sr2, so sr3 was linked to sk1 first. The remaining portion of sr3 was then linked to sk2 to maximize its reuse. Immediately afterward, sr1 and sr2 were linked to sk1 until both sources were minimized, as both have a shorter arm to sk1 than to sk2.

**Figure 4:**
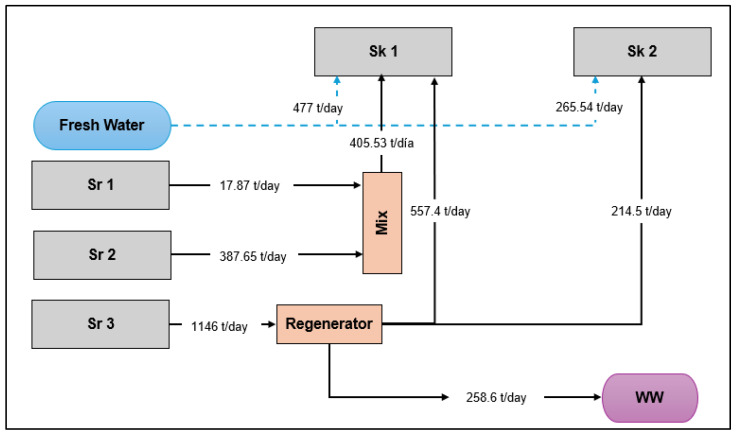
Mass reuse–recycling network for the integrated PVC suspension process using regeneration.

Figure 4 shows the water integration network for the PVC process, considering a PVA composition of 2.13 mgL^−1^ as the output concentration from the regenerator. It can be observed that sources sr1 and sr2 (405.5 t/day) were directly redirected to the polymerization reactor, sk1. The negligible PVA contents of these streams meant they could potentially be linked with both sk1 and sk2, with only the composition as limiting factor; therefore, the reaction section was prioritized for reuse as it is the main sink. For source 3, the stream went to the interceptor completely, as the concentration was significantly higher than the allowable limit for both sinks. From the system, a water stream of 771 t/day was recovered as permeate and was reused firstly in sk1 and then in sk2 at a 70:30 ratio, following the arm rule.

After establishing the water network, reductions of 61% and 83% were obtained for the freshwater intake and wastewater emissions, respectively. The freshwater for the reactor achieved reductions ranging from 1440 t/day to 477 t/day, while the boiler reduced its emissions to 480 t/day to 265.5 t/day. The reduction in freshwater is especially important, as it is higher compared to the savings achieved in studies such as that by Guardo-Ruiz et al. for similar PVC production processes, whereby direct reuse–recycling strategies obtained 21% freshwater savings and 26% wastewater reductions, with the boiler being the main target of the integration system [43]. The reduction achieved is particularly significant not only for the value but also because it impacted the reactor section’s freshwater intake. This sink requires a freshwater feed with a very stringent composition (demineralized water), which normally involves a higher cost as it is treated with several purification processes (filtration and ionic exchange membranes, etc.). Lima et al. found similar results for the optimization of a food processing plant (restrictive conditions) using a centralized regeneration system (rhizofiltration and ionic exchange resin), achieving 46% and 50% reductions in wastewater discharges and water intake, respectively [44].

From this same interceptor unit, there was a significant rejected flow of approximately 258.6 t/day (concentrated), which goes directly into the wastewater treatment plant for discharge. The remaining wastewater stream can be further reused for the additional sinks mentioned earlier (makeup water for cooling towers and water for general use), as they have no limitations in terms of composition or flow. In this scenario, the remaining wastewater streams are reduced almost to zero. However, including water from services may entail additional water flows and losses not addressed in this study [45]. A complete mapping of the water flow including utilities systems can identify potential sinks and sources, but at the same time requires an analysis of several contaminants and the effects on the sinks and on the regeneration systems [46].

**Figure 5:**
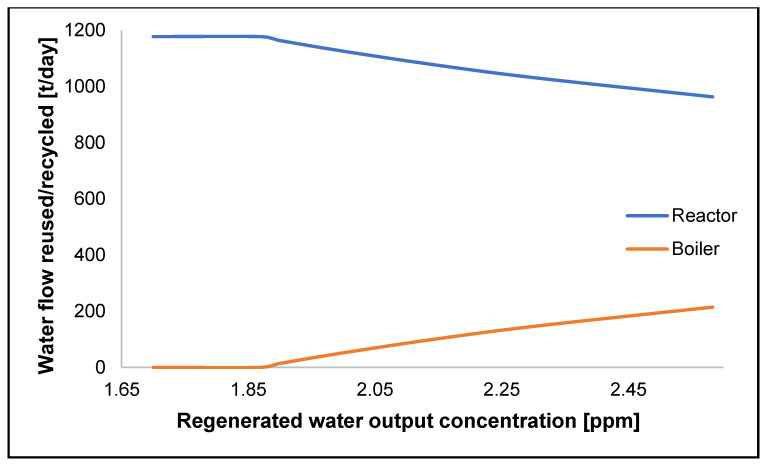
Water reused or recycled per sink compared to the regenerated water PVA concentration.

Figure 5 showcases the effect of the regeneration system’s efficiency as lower PVA concentrations are achieved and the distribution of the regenerated water is shifted towards the reactor if the linking of sr1 and sr2 with sk1 is maintained. The reactor section can take all the regenerated flow (771 t/day) plus wastewater from the recuperation of the VCM section (405 t/day) when the regeneration system achieves an output concentration of 1.86 ppm of lower. The complete water feed (1440 t/day) of the reaction section can dilute the contaminant concentration to permissible levels to use all the sources. On the other hand, at the same concentration, the boiler can only satisfy its requirements with freshwater.

### 3.2. Simulation of the Mass-Integrated Process

**Figure 6:**
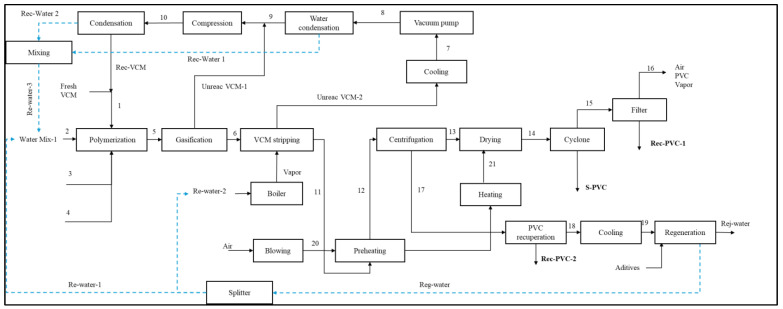
Flow diagram of the PVC production process integrated via regeneration.

Figure 6 shows the flow diagram of the PVC production process integrated mass-wise with regeneration, serving as a basis for simulation. The regeneration system allows the recovery of a sizable portion of the centrifuge water, which is subsequently sent to the reactor for the suspension formation. Additionally, water is recycled into the boiler, for the steam production system of the VCM separation stage. The streams from the VCM recovery system are linked to the reactor after being mixed into a single stream. On the other hand, two units for the recovery of residual PVC (filter and physicochemical treatment) were added, recovering around 13 tons per day. The energy integration of the process between the VCM stripping tower streams and the air for the dryer can also be observed. The reduction in this heat exchanger helps decrease the energy consumption of the heater (11 MJ/h).

**Figure 7:**
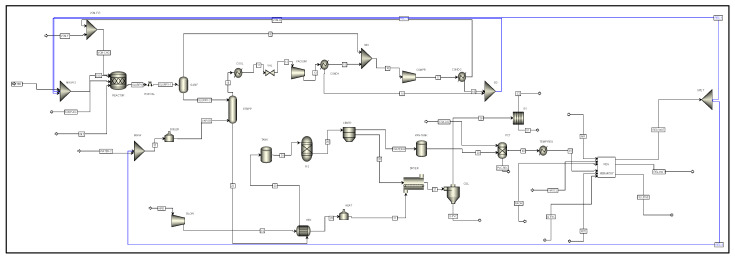
Flowsheet diagram of the mass- and energy-integrated PVC production process via regeneration.

Figure 7 show the flowsheet of the integrated PVC process through regeneration. The main fresh VCM stream (VCM-F) was mixed with a recirculated VCM stream (VCM-R) (80/20 ratio), and the resulting stream (VCM-CH) was fed into the polymerization reactor (REACTOR). The VCM reacted at a pressure of 10 kg-f × cm^−2^ and a temperature of 70 °C, within a suspension of water, PVA as a stabilizing agent (SUSP-AG), and the initiator (INIT). The water used in this stage was a mixture of streams from the regeneration system (REC-1), VCM recovery system (REC-2), and freshwater (WATER). At the end of the reaction, a mixture of solid PVC suspended in residual VCM within the suspension was obtained. The conversion of VCM to PVC caused a pressure change represented by a valve (EXP-VAL), which had an outlet pressure from the reactor of 3.5 kg-f × cm^−2^. To remove the VCM from the suspension, a gasification tank (GASIF) was used, where the pressure was reduced to 1.8 kg-f × cm^−2^, eliminating 95% of the unreacted VCM. The purged gas stream mainly contains the monomer; however, it also has a significant fraction of water (18 t/day) and small amounts of initiator and PVA (less than 1% for both).

The purged stream (SLURRY-2) in the liquid phase enters a stripping tower (STRIPP), where the residual VCM is reduced to less than 1 ppm. The residual VCM is eliminated by a high-pressure steam stream (14 kg-f × cm^−2^ and 225 °C) in the counterflow direction. The steam is generated from raw water in a boiler (BOILER) with a flow rate of 20 tons of steam per hour. The top stream enters the residual VCM recovery zone, where it is cooled in a cooler (COOL) to 50 °C. It then immediately enters a vacuum pump (VACUUM) that has an outlet pressure of 2.5 kg-f×cm^−2^. The stream is cooled in a condenser (COND-1) to 50 °C due to the temperature increase in the pump. Simultaneously, the residual VCM is separated from the water used for stripping. The purged VCM stream from the condenser and gasifier enters a compressor (COMPR), which raises the monomer’s pressure near its saturation pressure, allowing it to condense in a second condenser (COND-2) and be recirculated back to the start of the process at a pressure of 3.5 kg-f × cm^−2^ and temperature of 8 °C. Additionally, any remaining water fraction is purged in this step. According to the results from the mass integration, the water streams collected from the residual VCM recovery system are mixed and recirculated (REC-2) directly to the polymerization reactor due to their low contaminant content (insignificant traces).

The PVC slurry stream entered a heat exchanger (shell and tube), where its temperature was reduced to below 75 °C by transferring heat to an air stream used to remove the water in the dryer. The air stream (AIR) entered at 32 °C and reached a temperature of 91 °C. The heterogeneous mixture reduced its temperature to about 74 °C. For this unit, the HEATX model from the software was used, where the energy transfer was specified, along with the flow regime type, which in this case was counterflow. It was defined that the hot fluid was placed inside the shell (slurry). The PVC stream proceeded to the drying stage. In a centrifuge (CENTR), 75% of the water was removed, while a wet paste was formed. This unit generates the main wastewater stream (WATER-R) of the process, which contains a large portion of PVA and the initiator. The paste had a moisture content of 25% when entering the dryer (DRYER); the remaining moisture was removed using the air stream integrated at a temperature of 250 °C, heated by a burner (BURNER) with a flow rate of 6360 t/day. This stream left the polymer with a moisture content of 0.01% by weight. The product stream was introduced into the cyclone, as the dry polymer resin was contained in a mixture of hot gases, where the dry polymer particles were separated from the gaseous mixture. From this unit, the product stream of the dry granular polymer (S-PVC) with traces of monomer and water needed to maintain quality is produced. Also, a gas stream consisting of air, steam (stream 25), and some traces of polymer is ejected. The former was sent to a bag filter (BAG), which filtered 99% of the PVC particles carried by the gas mixture.

**Figure 8:**
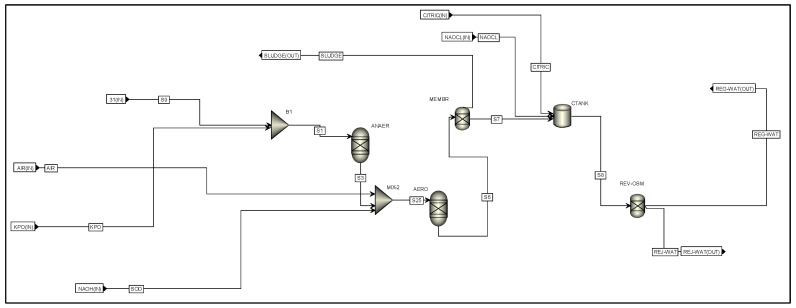
Flowsheet of the regeneration of the process effluents.

The wastewater stream (WATER-R) that exits the centrifuge was later introduced into the selected regeneration system, which consists of a physicochemical treatment (PCT) along with a regenerator (REG). The wastewater stream entered a tank where the physicochemical process takes place. This process includes flocculation, coagulation, and clarification of the remaining PVC particles in the water [47]. An aluminum coagulant (COA-AG) with an efficiency of 99% was used. The water was then cooled to a temperature of 35 °C before entering the regeneration system. Figure 8 shows the wastewater regeneration system from the centrifuge simulated in AspenPlus software. The system consists of two yield-type reactors, where changes in the stream compositions were specified. These tanks represent biological reactions to degrade PVA (anaerobic and aerobic). A series of substances, such as potassium oxide and air, are introduced into these tanks to facilitate these reactions. The ideal SEP model was used to simulate membrane separations, both in the reactor and the reverse osmosis tank. At the end of this system, regenerated water (REG-WAT) is produced under the conditions described in the integration section, along with rejected water (REJ-WAT). The regenerated stream is split and sent to the reactor and boiler for reuse. In comparison, for Gonzalez et al., the use of purges is recommended for streams that carry multiple contaminants when simulating water reuse–recycling networks (including via regeneration), reducing the potential savings in freshwater, as observed for a modified chitosan microbead production process with differences ranging between 30 and 40% [48].

### 3.3. WEP Analysis of the PVC Production Process Through Regeneration

Table 5 presents a summary of the parameters collected from the simulation used to quantify the indicators in the WEP analysis. During the data collection, the streams entering and leaving the process were considered, along with their composition and physical state (gas, liquid, or solid). These considerations allow for the proper classification of streams for the technical analysis. For example, water vapor was not considered as wastewater, and the PVC recovered in later stages, such as the bag filter or physicochemical treatment, was not considered as a final product stream. Additionally, the monomer was established as the sole raw material, since it is the only compound that undergoes a chemical transformation. On the other hand, the product flow generated was used as the basis for analyzing how efficient the process is in utilizing its mass and energy resources.

**Figure 9:**
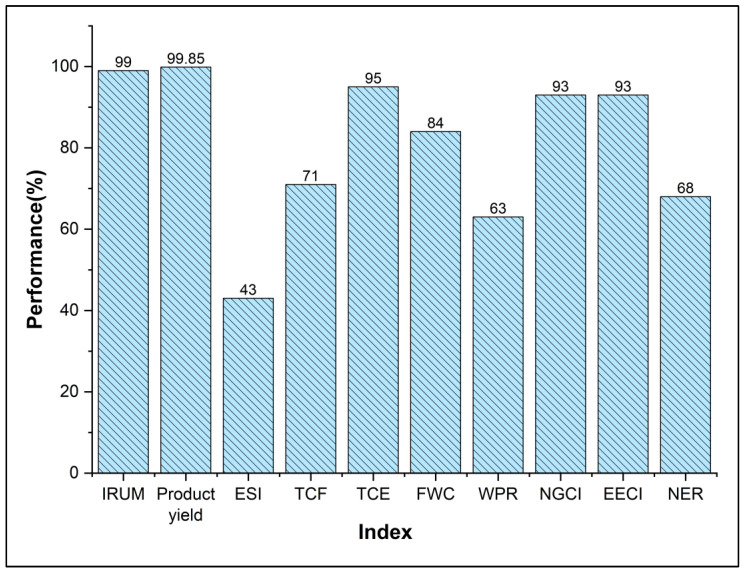
Performance of indicators for the PVC process integrated with energy and mass, including regeneration.

Figure 9 shows the performance of the evaluated indicators for the integrated mass and energy PVC production process. In the raw material usage indicators, it was found that the process is highly efficient. The production yield was 99.85%, and the product flow remained at 1150 t/day with a fresh VCM flow of 1152 t/day. This is expected for a technology with a significant maturation time, such as the suspension method [18]; similarly, hydrocracking units (mature technologies) present high production yields and use unconverted materials, with rates as high as 96% [49]. However, for the mass integration, two additional units were added to recover residual PVC from the cyclone top stream and from the centrifuge water. The total recovered mass was 13 t/day of PVC, showing a significant increase in process productivity. The recovered PVC is not considered a product due to its lower quality than the resin from the main product stream, meaning it is classified as a byproduct. On the other hand, the IRUM indicator remains the same in both scenarios, with 99% efficiency. The VCM recovery system helps reduce the consumption of fresh raw materials and helps mitigate emissions. The condenser and compressor system has a high recovery efficiency rate (288 t/day), although it uses a significant amount of energy (27% of the total).

On the other hand, the indicators related to water management were the most positively impacted by mass integration. For the integrated case, the FWC indicator achieved a performance rate of 84%. The regeneration of centrifuge effluent reduced the water consumption from 2.8 m^3^ per ton of PVC to 1.1 m^3^ per ton (for freshwater), leaving the process with a consumption rate lower than the 3.1 m^3^/t of the BAT (best available techniques) documentation [50]. However, this consumption only accounts for processing water and does not consider service water or losses. These additional sources can raise the index to exceed 3 m^3^ per ton of PVC. Reports such as those by PlasticsEurope indicate that the process may require 29.8 kg of service water per kg of PVC, significantly higher than the 2.7 kg of process water per kg of PVC [51]. This reduction in freshwater consumption has a positive economic impact on the process, as evidenced by the TCF indicator. The indicator achieved a performance of 71%, with the costs before integration amounting to $694,080 annually (for demineralized water only), compared to $267,322 annually for the integrated case. As expected, the regeneration approach had a higher impact in reducing the costs while decreasing freshwater withdrawals (can put pressure in ecosystems) than the direct reuse–recycling approach. However, these savings need to be contrasted in a more detailed economic analysis, as it is necessary to include the costs associated with the investment and operation of the regeneration system to assess its viability [52].

For the WPR indicator, a similar positive trend was observed as for the FWC indicator. The indicator rose to a value of 63%, from 8% before integration; as the reuse of process effluents was increased, less effluents were emitted. The reduction in the discharge of wastewater (originally 1565 t/day) not only mitigates potential environmental impacts from these sources (water scarcity or the emission substances) but also benefits the process economically, as less water is treated for release [53]. However, 258 tons of rejected water remains that should be utilized. The integration potential of the rejected water in the same analyzed sinks can be explored but those savings are not expected to be significant. Furthermore, mapping the whole water flow of the process offers a more complete image of the sinks that can benefit with the reuse of wastewater, such as utilities water systems (e.g., cooling tower losses), as those sinks are not constrained like the process sinks. An additional recovery system could be implemented to improve the quality to increase the reuse and recycling potential, making into a more decentralized system. Nonetheless, it is recommended to use advanced optimization methods to analyze all these alternatives (to find the optimal approach) under more rigorous considerations, such as handling multiple contaminants or with combined strategies or approaches, among others [54]. Additionally, it is advisable to compare other advanced effluent regeneration systems to identify networks with better economic performance.

Regarding the energy management of the process, the indicators showed uneven performances after integration. The ESI presented a value of 43%, with an energy consumption rate of 4.4 GJ/t of PVC. The inclusion of regeneration units significantly impacts the process’s energy consumption, accounting for 0.2 GJ/t of PVC of the total. Energy integration between the slurry stream and the air stream improves the process performance by reducing the energy consumption by 1.1 GJ/day; however, this represents only a 9% decrease, which may be insufficient. This increase necessitates a reconsideration of the energy integration to include new equipment where cooling or heating occurs. Strategies such as energy integration can mitigate potential issues related to pollutant emissions and safety risks. Additionally, the use of technologies such as heat pumps or cogeneration systems could facilitate the recovery of residual heat [55].

Similarly, the TCE reached a value of 95%, with a daily cost of $2188, which could be critical for the profitability of the process. The NGCI and EECI indicators provide a description of the distribution of energy sources for the process. For suspension production, natural gas is identified as the primary energy source (commonly accounting for approximately 93.8% of total energy) [56]. In the integrated process, 53.4 m^3^ of natural gas was used per ton of PVC, with the integration saving 6 m^3^ of fuel per ton of PVC. The predominant use of this energy source benefits the process compared to electricity use (which has a higher cost per unit) but may have negative environmental effects. Conversely, the electricity use in the integrated process was 2.4 kWh per ton of PVC, with the regeneration system significantly impacting this indicator. This demonstrates that the distribution of energy sources is affected, with 93% of the energy coming from natural gas and 7% from electricity in the integrated process.

Finally, the net energy ratio (NER) and energy usability index (EUI) indicators provide insight into the process’s energy utilization by analyzing the energy flow through material movement (inputs and outputs) and services. The NER obtained an efficiency rate of 68% from a 0.84 value. An NER greater than one indicates a positive balance between the energy entering and exiting the process. Considering this, the process performance was acceptable compared to the best case of 1, meaning the process manages its high volume of energy quite well but there is still room for improvement. The NER decreased compared to the 0.9 value from Mendivil et al.’s study, but it was an improvement from the value of 0.7 before mass integration [29]. On the other hand, the EUI followed the same trend as the NER, with a value of 3.99 for the process. The energy contents of the polymer and monomer were high but similar (HHV = 18 MJ/kg for PVC and HHV = 16 MJ/kg for the monomer), with the difference being driven by the increased energy consumption of the process. Regardless, PVC remains a product that should be maximized through circular economic strategies whenever possible, as it is a valuable product from an energy standpoint.

## 4. Conclusions

In this study, mass integration using a regeneration approach was applied to the suspension PVC process. The reuse–recycling system’s performance was evaluated using the WEP analysis. The process achieved a 61% saving of freshwater with an 83% reduction in wastewater emissions. At the same time, 258.6 t/day of residual wastewater remained, and the inclusion of more sinks with loose restrictions can help further reuse these effluents. The approach of fully treating above-limit streams achieved better results than only partially recycling them directly. The simulation of the integrated process through AspenPlus served as source of data regarding the process performance. The technical analysis showed that the regeneration approach substantially benefits the performance of the process, as the water management indicators improve, alongside significant freshwater cost reductions. However, it required additional energy inputs to operate as the ESI fell below 50%. Regardless, the NER showed good energy management, achieving a 0.84 value. Nonetheless, this solution may not be the most optimal for the process. A mathematical approach could be used to pinpoint it, as a combined approach could be assessed considering other regeneration systems, as well as more contaminants; additionally, an in-depth economic assessment could be implemented.

## Figures and Tables

**Table 1:** Comparison of the literature findings on mass integration and technical evaluation cases for processes related to PVC production.

Process	Mass Integration	Water Regeneration	WEP Technical Evaluation	Reference
PVC Manufacturing	Yes	No	No	[20]
Sodium Hydroxide and Suspension PVC Plant	Yes	No	No	[21]
PVC Production (Batch Plant)	Yes	No	No	[22]
Energy-Integrated Suspension PVC Production Process	Yes	Yes	Yes	This work

**Table 2:** WEP indicators for the technical evaluation of chemical processes.

Variable	Units	Equation
Production Yield	%	$\gamma_{i}=\frac{mass\;flow\;of\;product}{mass\;flow\;of\;main\;feedstock}\times 100\%$
Fractional Water Consumption (FWC)	m^3^/t	$FWC=\frac{volume\;flow\;of\;freshwater}{mass\;flow\;of\;product}$
Total Cost of Freshwater (TCF)	$/day	TCF=flowrate of freshwater consumed×cost of freshwater
Wastewater Production Ratio (WPR)	%	$WPR=\frac{wastewater\;volumetric\;flow\;}{freshwater\;volumetric\;flow}\times 100\%$
Index of Reused Unconverted Material (IRUM)	%	$IRUM=\frac{reused\;material\;i\;mass\;flow}{unconverted\;material\;i\;mass\;flow}\times 100\%$
Total Cost of Energy (TCE)	$/day	TCE=total energy consumed×cost of energy
Energy Specific Intensity (ESI)	MJ/t	$R_{ESI}=\frac{total\;energy\;consumed\;}{product\;mass\;flow}$
Net Energy ratio (NER)	Dimensionless	$NER=\frac{\;product\;calorific\;power\times product\;mass\;flow}{total\;consumed\;energy+(feedstock\;calorific\;power\times feedstock\;mass\;flow)}$
Energy Usability Index (EUI)	Dimensionless	$EUI=\frac{product\;calorific\;power\times product\;mass\;flow}{total\;consumed\;energy}$
Natural Gas Consumption Index (NGCI)	m^3^/t	$NGCI=\frac{total\;natural\;gas\;consumed}{product\;mass\;flow}$
Electric Energy Consumption Index (EECI)	kWh/t	$EECI=\frac{total\;electricity\;consumed}{product\;mass\;flow}$

**Table 3:** Sources of the PVC suspension process.

Source	Mass Flow [t/day]	Water Flow [L]	PVA [mgL^−1^]	Mass Fraction	Load [t/day]	Origin
sr1	17.87	17,929.7	0	0	0	Condenser 1
sr2	442.5	443,862	0	0	0	Condenser 2
sr3	1104.8	1,104,228.8	1173.7	0.00117	1292	centrifuge

**Table 4:** Sinks of the PVC suspension process.

Sink	Mass Flow [t/day]	Water Flow [L]	PVA [mgL^−1^]	Mass Fraction	Load [t/day]	Destination
sk1	1440	1,444,332.999	1	1.00301 × 10^−6^	0.0014	reactor
sk2	480	481,444.333	10	1.00301 × 10^−5^	0.0048	Boiler

**Table 5:** WEP analysis parameters for the mass- and energy-integrated PVC production process.

Parameter	Unit	Value
Mass flow of raw material (VCM)	t/day	1152
Mass flow of recycled raw material	t/day	288
Mass flow of unreacted raw material	t/day	288
Mass flow of product	t/day	1150
Total volumetric flow of freshwater	m^3^/day	746
Total volumetric flow of wastewater	m^3^/day	279
Total energy consumption	GJ/day	4912.63

## Data Availability

The data that support the findings of this study are available from the corresponding author, Á.D.G.-D., upon reasonable request.
